# Factors affecting match running performance in elite soccer: Analysis of UEFA Champions League matches

**DOI:** 10.5114/biolsport.2023.116453

**Published:** 2022-06-01

**Authors:** Toni Modric, Sime Versic, Marko Stojanovic, Paweł Chmura, Marcin Andrzejewski, Marek Konefał, Damir Sekulic

**Affiliations:** 1 Faculty of Kinesiology, University of Split, Split, Croatia; 2 HNK Hajduk, Split, Croatia; 3 Faculty of Sport and Physical Education, University of Novi Sad, Novi Sad, Serbia; 4 Department of Team Games, Wrocław Univeristy of Health and Sport Sciences, Wrocław, Poland; 5 Department of Methodology of Recreation, Poznań University of Physical Education, Poznań, Poland; 6 Department of Human Motor Skills, Wrocław University of Health and Sport Sciences, Wrocław, Poland

**Keywords:** Football, Physical demands, Situational variables, Contextual factors, Linear mixed models

## Abstract

This study aimed to examine the independent effect of different match-related factors on match running performance (MRP) in elite soccer. Players’ MRPs (n = 244) were collected during UEFA Champions League (UCL) group stage matches in the 2020–21 season. All MRP data were collected by the semi-automatic optical system InStat Fitness (InStat Limited, Limerick, Republic of Ireland). Match-related factors included match outcome, team quality, match location, opponent quality and difference in team quality, while MRP included cumulative and relative measures of total distance (TD and R-TD), low-intensity running (LIR and R-LIR) (≤ 4 m/s), moderate-intensity running (MIR and R-MIR) (4–5.5 m/s) and high-intensity running (HIR and R-HIR) (≥ 5.5 m/s). Linear mixed models were used to examine the collective effect of match-related factors on MRPs when controlling for between-player, between-playing position and between-team variation. The main findings were that match outcome was associated with reduced HIR (d = -0.38, p = 0.04), match location was associated with increased TD, R-TD, LIR and R-LIR (d = 0.54–0.87, all p < 0.01), while team quality, opponent quality and difference in team quality were not associated with MRP. These results show that (i) winning UCL matches was not strongly influenced by players’ physical performance, (ii) away UCL matches were characterized by a slower match pace and greater match volume, and (iii) players’ physical performance was similar irrespective of playing either in or against high- or low-quality teams. The findings from this study may help soccer coaches to ensure optimal physical preparation of players in elite soccer.

## INTRODUCTION

Soccer is a complex team sport characterized by high physical demands [1, 2]. To understand such demands, match running performance (MRP) is commonly analysed by quantification of the total distance covered, distances covered in various speed zones and acceleration rates [3–6]. Although MRP is highly variable due to the influence of different match-related factors [7, 8], there is a general consensus that soccer players during the match can cover between 9 and 14 km and can perform 0.7–3.9 km of high-speed distance, 0.2–0.6 km of sprint distance and approximately 600 accelerations [9, 10].

These findings undoubtedly have developed an understanding of the physical demands of soccer; however, a plethora of studies have analysed the effects of match-related factors on match running performance, and these metrics should not be analysed in isolation [11]. Indeed, empirical evidence suggests that factors such as match location (i.e., playing at home or away), team quality and opposition quality (i.e., defined by the final ranking at the end of the league or season coefficients) or match outcome (i.e., win, loss or draw) strongly affect MRP [12]. However, although the association between MRP and different match-related factors is well documented, previous studies have reported inconsistent findings [7].

In brief, older studies demonstrated that soccer players performed significantly fewer high-intensity activities when winning than when losing [12, 13], while more recent studies reported no differences in distance covered at higher speeds regardless of the match outcome [9, 14]. There was a similar inconsistency among studies that analysed the association between MRP and match location. For example, although both belong to a similar competition level, Spanish players accumulated significantly greater total distance during away matches [14], while Portuguese players covered significantly more total distance at home matches [9]. A lack of consistency also was observed when analysing MRP and team quality. Specifically, while less successful teams (i.e., bottom ranked in competition) from the English Premier League covered a greater high-intensity distance (above 19.8 km/h and 25.2 km/h) than successful teams [15], both successful and unsuccessful teams from the Spanish La Liga presented the same RPs at higher velocities (above 14 km/h, 21 km/h and 24 km/h) [16]. Also, it is reported that MRP was higher when teams competed against higher-ranked opponents than against lower-ranked opponents [17]. Yet, some studies did not find significant differences in MRP between matches played against low-, medium- and high-ranked opponents [14], while other studies reported even greater MRP against lower-ranked competition [18].

Considering a very recent study which suggested that MRP may be influenced by multiple variables [19], it may be difficult to generalize associations between MRP and match-related factors. Furthermore, it should be emphasized that most of the previously conducted studies investigated such factors in isolation, and did not adequately control for the wide range of confounders. The application of an analysis that intends to assess the interaction effect, as well as the control of confounder variables, may bridge this research gap. Finally, although it was recently suggested that additional research should be conducted to develop an understanding of the influence of match-related factors on MRP across different leagues and countries [9], no study has analysed how match-related factors affect MRP in an elite soccer competition – the UEFA Champions League (UCL). Therefore, this study aimed to examine the independent effect of different match-related factors (match outcome, team quality, match location and opponent quality) on MRP in UCL matches.

## MATERIALS AND METHODS

### Participants

Match running performance (n = 224) of elite soccer players (n = 179) from teams (n = 24) that competed in the group stage of UCL during the 2020–21 season were included as cases in this study. Participants were classified as “elite” as the UCL consists of the best teams from European national competitions. Teams can qualify for the UCL directly (i.e., based on the final position in national competitions) or through qualification rounds.

All data were randomly obtained from 20 matches from groups A (n = 3), B (n = 3), C (n = 4), E (n = 4), F (n = 3) and G (n = 3). Players were classified by match playing position using official match reports provided by InStat Scout (InStat Limited, Limerick, Republic of Ireland) as: central defenders (CD), fullbacks (FB), central midfielders (CM), wide midfielders (WM) and forwards (FW) [20]. Goalkeepers were excluded from the cohort due to the uniqueness of the position. Only the MRPs of those players who played in whole matches were analysed [21]. As a result, the average number of observations for each participant was 1.36. The research was approved by the ethical board of the Faculty of Kinesiology, University of Split.

### Procedures and data collection

MRP data were collected using a semi-automatic multiple-camera system, InStat Fitness (InStat Limited, Limerick, Republic of Ireland). This optical system has a sampling frequency of 25 Hz, and identifies players by their movement, shape, and colour information. The reliability of the system has been demonstrated by comparison of data to the Vicon system (Vicon Motion Systems, Oxford Metrics, UK) at five velocity bands (0–7 km/h, 7–15 km/h, 15–20 km/h, 20–25 km/h, and 25+ km/h). This procedure included analysis of mean velocity difference compared to Vicon (m/s) and mean position difference (m) compared to Vicon, and the system has passed the official Fédération Internationale de Soccer Association (FIFA) test protocol for Electronic and Performance Tracking Systems (EPTS) (authorization number: 1007382). A detailed report is available on the official FIFA webpage [22]. Finally, previous research showed that this system is very accurate with high levels of absolute and relative reliability [23].

Classification of MRP was similar to other studies which used the same thresholds [15, 17], although some exceptions should be noted. Specifically, since it is generally accepted that walking (0.2–2 m/s) and jogging (2–4 m/s) are not crucial for successful match performances [15], we considered it as one zone (i.e., low-intensity running). On the other hand, high-intensity running (i.e., high speed running (5.5–7 m/s) + sprinting (≥ 7 m/s) is typically considered as a vital element of success in soccer [15], and therefore we believed that it might be of greater interest to observe it as one zone. As result, MRP variables in our study included total distance covered (TD) (m), low-intensity running (LIR) (≤ 4 m/s) (m), moderate-intensity running (MIR) (4–5.5 m/s) (m) and high-intensity running (HIR) (≥ 5.5 m/s) (m), relative total distance covered (R-TD) (m/min), relative low-intensity running (R-LIR) (≤ 4 m/s) (m/min), relative moderate-intensity running (R-MIR) (4–5.5 m/s) (m/min) and relative high-intensity running (R-HIR) (≥ 5.5 m/s) (m/min).

Match-related factors in this study included match outcome, team quality, match location and opponent quality. Match outcome was assessed as won or not won (i.e., loss or draw) and match location as playing at home or away [24]. Team quality, opponent quality and differences between them were evaluated using UEFA season club coefficients [25]. The season club coefficients are based on the results of clubs competing in the current UEFA Champions League, UEFA Europa League and UEFA Europa Conference League season. Clubs’ coefficients are determined either as the sum of all points won in the previous five years OR the association coefficient over the same period, whichever is higher. Points awarded each season are in accordance with the relevant competition regulations for that specific season [26].

### Statistical analysis

Before running linear mixed effect models, assumptions of independence and collinearity were checked, and box plots and histograms were used to determine potential influential data points. Following analysis, visual inspections of residual plots were used to determine obvious deviations from homoscedasticity or normality [27]. Afterward, a linear mixed model was used to examine the independent influence of the match-related factors on MRP. In this study, eight separate models were developed to examine the influence of fixed effects (match outcome, team quality, match location, opponent quality, difference in team quality) and random effects (team identity, playing position, player identity) on TD, R-TD, LIR, R-LIR, MIR, R-MIR, HIR, and R-HIR (dependent variables). Match outcome and match location were introduced as dummy variables, while team quality and opponent quality were continuous variables. Random factors were included in the model to allow random deviations for playing positions, teams and players from the overall fixed intercept and fixed coefficients (Table 1).

**TABLE 1:** Model specification.

Level of data		Factors	Type	Classification
Level 4	Cluster of clusters (random factor)	*Team*		

Level 3	Cluster of clusters (random factor)	*Position*		

Level 2	Cluster of units (random factor)	*Player*		

Level 1	Unit of analysis	*Individual match observations*		
	Dependent variables	Accumulative total distance (Model 1)	Continuous	meter (m)
		Accumulative low intensity running (Model 2)	Continuous	meter (m)
		Accumulative moderate intensity running (Model 3)	Continuous	meter (m)
		Accumulative high intensity running (Model 4)	Continuous	meter (m)
		Relative total distance (Model 5)	Continuous	meter per min (m/min)
		Relative low intensity running (Model 6)	Continuous	meter per min (m/min)
		Relative moderate intensity running (Model 7)	Continuous	meter per min (m/min)
		Relative high intensity running (Model 8)	Continuous	meter per min (m/min)

	Covariates	Match outcome	Dummy variable	0 = Loss/Draw 1 = Win
		Team quality	Continuous	UEFA club coefficient
		Location	Dummy variable	0 = Home 1 = Away
		Opponent quality	Continuous	UEFA club coefficient
		Difference in team quality	Continuous	UEFA club coefficient

A ‘step-up’ model construction strategy was employed, similar to that used in the previous team sports mixed model studies [27, 28]. The modelling began with an ‘unconditional’ model using only a fixed intercept and random factors. The model was further developed by adding each single fixed effect. The order in which each fixed effect was added to the model was guided by a senior performance staff member based on extensive experience in the field [27]. The importance of each fixed effect was determined by whether its inclusion demonstrated statistically significant (p < 0.05, likelihood ratio test method [29]) improvements compared to the explanatory model. Also, the Akaike information criterion (AIC) and degrees of freedom (df) for each model were visually compared to the previous model, where a lower AIC represented a better model. The t statistics from the mixed models were converted into Cohen’s d effect sizes and interpreted as < 0.2 trivial; 0.2–0.6 small; 0.7–1.2 moderate; 1.2–2.0 large; > 2.0 very large [27, 30]. All statistical analyses were conducted using SPSS software (IBM, SPSS, Version 25.0).

## RESULTS

The effects of the different match-related factors on MRP are presented in Tables 2, 3, 4, 5. For all models, the best fit for the data was found by including random intercepts for each player, team and playing position. Introducing random slopes to the models in this study did not yield a better model fit (no additional lowering of AIC). The results showed that players covered greater TD (t = 2.69, p < 0.01, d = 0.54) and achieved greater R-TD (t = 2.57, p = 0.012, d = 0.55) in away matches. None of the other factors of interest contributed to a significantly different or better fit of Models 1 and 2 (all p > 0.05) (Table 2). LIR (t = 3.50, p < 0.001, d = 0.67) and R-LIR (t = 4.10, p < 0.001, d = 0.87) were also greater when playing away. Match outcome, team quality, opponent quality and difference in team quality had no effect on LIR and R-LIR (all p > 0.05) (Table 3). No associations between match-related factors, and MIR and R-MIR were found (all p > 0.05) (Table 4). Players covered lower HIR in winning matches (t = -2.06, p = 0.04, d = -0.38). Match location, team quality, opponent quality and difference in team quality did not contribute to a significantly different or better fit of Model 7 (all p > 0.05). In addition, no associations between match-related factors and R-HIR were found (all p > 0.05) (Model 8) (Table 5).

**TABLE 2:** Influence of contextual factors on accumulative total distance (Model 1) and relative total distance (Model 2).

Fixed effects	Coefficients	95% CI	SE	df	t value	p	AIC difference	Likelihood ratio test	Effect size (95% CI)
Model 1									
(Intercept)	10833.2	10037.5; 11628.9	299.9	4.5	36.12	0.001			
Match outcome	NS								
Team quality	NS								
Match location	197.2	51.7; 342.8	73.3	100.4	2.69	0.008	17	< 0.001	0.54 (0.14; 0.93)
Opponent quality	NS								
Difference in team quality	NS								

Model 2									
(Intercept)	111.7	103.5; 119.9	3.30	5.61	33.82	0.001			
Match outcome	NS								
Team quality	NS								
Match location	1.74	0.4; 3.1	0.68	86.56	2.57	0.012	8	0.004	0.55 (0.12; 0.98)
Opponent quality	NS								
Difference in team quality	NS								

CI: confidence interval; SE: standard error; df: degrees of freedom; AIC: Akaike information criterion. Note: Likelihood ratio test based on comparison with previous model. Effect size is an approximation based on the t value obtained from each variable included in the model. NS: covariate not significant in final model.

**TABLE 3:** Influence of contextual factors on accumulative low-intensity running (Model 3) and relative low-intensity running (Model 4).

Fixed effects	Coefficients	95% CI	SE	df	t value	^p^	AIC difference	Likelihood ratio test	Effect size (95% CI)
Model 3									
(Intercept)	7996.1	7739.1; 8253.1	110.2	7.5	72.55	0.001			
Match outcome	NS								
Team quality	NS								
Match location	193.3	83.9; 302.8	55.2	110.3	3.50	0.001	20	< 0.001	0.67 (0.28; 1.05)
Opponent quality	NS								
Difference in team quality	NS								

Model 4									
(Intercept)	81.61	78.70; 84.53	1.35	14.41	59.95	0.001			
Match outcome	NS								
Team quality	NS								
Match location	2.00		0.49	88.48	4.10	0.001	15	< 0.001	0.87 (0.43; 1.31)
Opponent quality	NS								
Difference in team quality	NS								

CI: confidence interval; SE: standard error; df: degrees of freedom; AIC: Akaike information criterion. Note: Likelihood ratio test based on comparison with previous model. Effect size is an approximation based on the t value obtained from each variable included in the model. NS: covariate not significant in final model.

**TABLE 4:** Influence of contextual factors on accumulative moderate-intensity running (Model 5) and relative moderate-intensity running (Model 6).

Fixed effects	Coefficients	95% CI	SE	df	t value	p	AIC difference	Likelihood ratio test	Effect size (95% CI)
Model 5									
(Intercept)	1920.1	1469.3; 2370.9	165	4.17	11.63	0.001			
Match outcome	NS								
Team quality	NS								
Match location	NS								
Opponent quality	NS								
Difference in team quality	NS								

Model 6									
(Intercept)	20.1	15.4; 24.8	1.73	4.20	11.60	0.001			
Match outcome	NS								
Team quality	NS								
Match location	NS								
Opponent quality	NS								
Difference in team quality	NS								

CI: confidence interval; SE: standard error; df: degrees of freedom; AIC: Akaike information criterion. Note: Likelihood ratio test based on comparison with previous model. Effect size is an approximation based on the t value obtained from each variable included in the model. NS: covariate not significant in final model.

**TABLE 5:** Influence of contextual factors on accumulative high-intensity running (Model 7) and relative high-intensity running (Model 8).

Fixed effects	Coefficients	95% CI	SE	df	t value	^p^	AIC difference	Likelihood ratio test	Effect size (95% CI)
Model 7									
(Intercept)	948.9	729.9; 1167.9	83.1	4.6	11.42	0.000			
Match outcome	-61.8	-121.3; -2.3	30.0	116	-2.06	0.042	13	< 0.001	-0.38 (-0.75; -0.01)
Team quality	NS								
Match location	NS								
Opponent quality	NS								
Difference in team quality	NS								

Model 8									
(Intercept)	9.70	7.4; 12.0	0.87	4.52	11.18	0.001			
Match outcome	NS								
Team quality	NS								
Match location	NS								
Opponent quality	NS								
Difference in team quality	NS								

CI: confidence interval; SE: standard error; df: degrees of freedom; AIC: Akaike information criterion. Note: Likelihood ratio test based on comparison with previous model. Effect size is an approximation based on the t value obtained from each variable included in the model. NS: covariate not significant in final model.

**FIG. 1:**
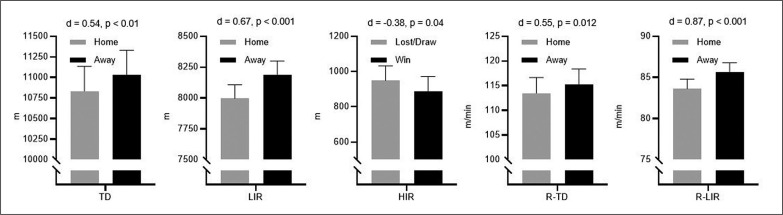
The key descriptive data from this study (data are given as Mean ± SE).

The key descriptive data from this study are presented in Figure 1.

## DISCUSSION

This study was the first to investigate the collective effect of different match-related factors on MRP when controlling for between-player, between-playing position and between-team variation in UCL. The main findings were that (i) match outcome was associated with reduced HIR; (ii) match location was associated with increased TD, R-TD, LIR, and R-LIR; (iii) team quality, opponent quality and difference in team quality were not associated with MRP.

Our results did not reveal an association between TD, R-TD, LIR, R-LIR, MIR or R-MIR and match outcome, suggesting that the final scores in UCL matches were not determined by overall distance or the distance covered at low or moderate speeds. On the other hand, there was a significant association (small effect size) between HIR (≥ 19.8 km/h) and match outcome. Specifically, in UCL match wins, players had a lower amount of HIR, indicating that successful UCL matches result in less cumulative total HIR. Conversely, when losing or drawing, elite soccer players had greater HIR, possibly to recover from an unfavourable match score [8, 31]. Nevertheless, it must be emphasized that no effect of match outcome on R-HIR was witnessed, confirming that successful match outcomes in the UCL were not associated with increased HIR. All these findings collectively demonstrate that winning UCL matches was not strongly influenced by players’ physical performance. The high degree of technical and tactical capabilities of the players likely plays a crucial role in winning matches in elite soccer. Such a consideration supports the idea that, in modern soccer, overall technical and tactical effectiveness probably has a greater impact on results than MRP [16, 32].

Elite soccer players covered similar MIR and HIR distances, and achieved similar R-MIR and R-HIR irrespective of whether the matches were played at home or away. On the other hand, increased TD, R-TD, LIR and R-TD were associated with match location (all small to moderate effect sizes). Specifically, greater overall cumulative and relative distance and distance covered at lower speeds (e.g., walking and jogging) were observed at UCL matches that were played away. Although it is generally accepted that low-intensity activities, such as walking and jogging, are not crucial in elite soccer performance [15], such activities can provide a better understanding of the pace that players experience during the match. Thus, the greater LIR and R-LIR in our study suggest that away UCL matches were characterized by a slower match pace. Slower performances during away matches may have been a consequence of the tactical approaches and defensive strategies adopted to limit the opposition rather than using an attacking threat [9, 33]. The possible defensive strategies adopted during away matches most likely resulted in greater TD and R-TD as well. According to the findings of a contemporary study, teams that apply this approach (i.e., playing defensively without high ball possession) generally cover a greater overall distance, especially at low and medium speeds [20]. However, as we did not analyse playing strategies, these considerations should be confirmed in future studies by including technical and tactical factors in the analyses.

Results from our study did not indicate an effect of opponent quality on MRP in UCL matches. Specifically, MRPs were similar irrespective of playing against high- or low-quality teams. Such findings suggest that elite soccer players’ physical performance was stable whether playing against “better” or “weaker” opponents. Taking into account the previous idea that professional soccer players regulate their physical efforts according to the specific demands of the individual matches [12, 17], these results may seem surprising. However, we believe that our findings are driven by the specific nature of the observed competition (i.e. UCL). Specifically, the UCL is an elite soccer club competition played by the world’s best soccer players [21]. Such players are highly professional athletes whose match performance is most likely consistently at a high level, which almost certainly resulted in stable physical performance irrespective of their opponents. Possibly, for similar reasons (i.e., the high standard of the UCL players) no effects of team quality or difference in team quality on MRP in UCL matches were found. Namely, our results indicated that MRP was not associated with these team-quality indicators, meaning that players’ physical performance was similar (i.e., stable) irrespective of the quality of their teams and regardless of the differences in the quality of the opponent teams. Considering previous studies which investigated MRP in UCL [21, 34, 35], such findings are actually expected. The results from these studies imply that UCL is a highly physically demanding competition, and therefore to compete at elite soccer all UCL players must be able to handle high physical demands. This likely results in high (i.e., similar) MRP during the matches for all players and teams.

The present investigation has some limitations that should be considered. First, this study did not analyse all matches from the group stage of the UCL. Specifically, only 20 selected matches were observed. However, this is a very common obstacle in studies involving players who compete in elite soccer [36, 37]. For methodological reasons we included only players who played the whole match, which reduced the number of observations and may have affected MRP. In addition, we did not observe MRP in all speed zones. Future research should consider other match-related factors, such as weather conditions, team formations, match periods, current score, match importance, and number of players on the field, which have all been confirmed to influence MRP [19, 24]. Future research should analyse other MRP metrics such as accelerations/decelerations and metabolic power, considering peak running outputs. Finally, for a detailed understanding of the effect of match-related factors on running performance in UCL, external training load and its relationship with match loads should also be considered in future research [38].

## CONCLUSIONS

Winning UCL matches was not strongly influenced by players’ physical performance. Specifically, successful match performance (i.e., win) results in lower cumulative total HIR, possibly indicating that a high degree of technical and tactical capabilities of the players plays a crucial role in winning matches in elite soccer. However, it is more important to emphasize that unsuccessful match performance (i.e., loss and draw) in UCL results in greater HIR. As greater HIR is associated with greater blood lactate accumulation [39], different recovery strategies after UCL losses and draws should be applied than after wins.

Although playing away was associated with increased TD, R-TD, LIR and R-LIR, which clearly indicates that away matches were characterized by a slower match pace and greater match volume, the overall effect of match location on MRP is limited. Specifically, there is no effect of match location on HIR, which is considered as one of the vital elements of soccer [15]. However, as observed matches were played without an audience or with limited capacity in the stands due to the COVID-19 pandemic [40], the effect of match location should be further investigated.

Finally, there was also no effect of any of the team quality indicators on MRP. As UCL is a very physically demanding competition [21, 34, 35], this most likely indicates that players’ physical performance was high irrespective of playing either in or against high- or low-quality teams. Aiming to handle such high physical demands, soccer coaches should ensure an adequate conditioning stimulus in preparation for every match in UCL. Ultimately, such an approach may help coaching staff in their efforts to ensure optimal physical preparation of players who participate in elite soccer and who are seeking UCL qualification.
